# Targeting STAT3 Signaling in COL1+ Fibroblasts Controls Colitis-Associated Cancer in Mice

**DOI:** 10.3390/cancers14061472

**Published:** 2022-03-14

**Authors:** Christina Heichler, Anabel Schmied, Karin Enderle, Kristina Scheibe, Marta Murawska, Benjamin Schmid, Maximilian J. Waldner, Markus F. Neurath, Clemens Neufert

**Affiliations:** 1 First Department of Medicine, Friedrich-Alexander-Universität Erlangen-Nürnberg, Universitätsklinikum Erlangen, 91054 Erlangen, Germany; christina.heichler@gmx.de (C.H.); anabel_schmied@gmx.de (A.S.); karin.enderle@uk-erlangen.de (K.E.); kristina.scheibe@uk-erlangen.de (K.S.); marta.murawska@uk-erlangen.de (M.M.); maximilian.waldner@uk-erlangen.de (M.J.W.); markus.neurath@uk-erlangen.de (M.F.N.); 2 Optical Imaging Centre Erlangen (OICE), University of Erlangen-Nürnberg, 91058 Erlangen, Germany; benjamin.schmid@fau.de; 3 Deutsches Zentrum Immuntherapie (DZI), Universitätsklinikum Erlangen, University of Erlangen-Nürnberg, 91052 Erlangen, Germany

**Keywords:** fibroblast, collagen, colorectal cancer, tumorigenesis, AOM/DSS model, inflammation

## Abstract

**Simple Summary:**

Colitis-associated cancer is a colorectal cancer entity with poor prognosis and limited therapeutic options typically occurring as long-term complications of inflammatory bowel diseases. Connective tissue cells such as cancer-associated fibroblasts are part of the tumor microenvironment that can influence cancer development. The aim of this study was to determine the role of STAT3 activation in a frequent subset of fibroblasts during the development of inflammation-associated colorectal cancer in vivo. Our work highlights the functional role of cancer-associated fibroblasts in colitis-associated cancer, suggesting that strategies targeting the activation of that cell type could evolve as promising therapeutic option in inflammation-associated colorectal cancer and possibly additional entities.

**Abstract:**

Colorectal cancer (CRC) is a common disease and has limited treatment options. The importance of cancer-associated fibroblasts (CAFs) within the tumor microenvironment (TME) in CRC has been increasingly recognized. However, the role of CAF subsets in CRC is hardly understood and opposing functions of type I (COL1+) vs. type VI (COL6+) collagen-expressing subsets were reported before with respect to NFκB-related signaling. Here, we have focused on COL1+ fibroblasts, which represent a frequent CAF population in CRC and studied their role upon STAT3 activation in vivo. Using a dual strategy with a conditional gain-of-function and a conditional loss-of-function approach in an in vivo model of colitis-associated cancer, tumor development was evaluated by different readouts, including advanced imaging methodologies, e.g., light sheet microscopy and CT-scan. Our data demonstrate that the inhibition of STAT3 activation in COL1+ fibroblasts reduces tumor burden, whereas the constitutive activation of STAT3 promotes the development of inflammation-driven CRC. In addition, our work characterizes the co-expression and distribution of type I and type VI collagen by CAFs in inflammation-associated colorectal cancer using reporter mice. This work indicates a critical contribution of STAT3 signaling in COL1+ CAFs, suggesting that the blockade of STAT3 activation in type I collagen-expressing fibroblasts could serve as promising therapeutic targets in colitis-associated CRC. In combination with previous work by others and us, our current findings highlight the context-dependent roles of COL1+ CAFs and COL6+ CAFs that might be variable according to the specific pathway activated.

## 1. Introduction

Colorectal cancer (CRC) is an ongoing medical challenge with a clinical course of disease that can vary markedly between different individuals. The etiology of CRC is multifactorial and includes hereditary and environmental drivers/parameters [1]. Colitis-associated cancer is a subtype of CRC developing in individuals suffering from inflammatory bowel diseases such as ulcerative colitis and Crohn’s disease [2]. Some genetic aberrations in tumor epithelial cells (such as RAS, BRAF, and MMR/MSI) are well-accepted factors with prognostic and predictive value in CRC guiding clinical decision making, including the selection of the therapeutic armamentarium for an individual patient [1]. Moreover, additional factors such as hypoxia in the tumor microenvironment (TME) are considered to influence the course of tumor development and chemotherapy resistance in CRC and other tumor entities [3,4]. In fact, CRC consists not only of tumor epithelial cells, but the cancer tissue also comprises extracellular matrix proteins and stromal cells including various immune and nonimmune cells, which altogether form TME. Contributions from TME during the pathogenesis of CRC have become a major scientific focus more recently. In fact, TME has been suggested to influence critical pathobiological steps during the development of CRC [5,6,7,8]. From a clinical perspective, stromal gene expression was shown to define poor-prognosis subtypes in CRC [9,10]. In line with that, a consensus molecular classification (CMS) of CRC proposed four major subtypes based on gene expression patterns [11]. The CMS4 subset with a mesenchymal kind of signature including elevated angiogenesis, stromal invasion and transforming growth factor beta activation exhibited the worse relapse-free and overall survival [11]. These observations highlighted the potential clinical importance of mesenchymal cells such as cancer-associated fibroblasts (CAFs), which are major constituents of tumor stroma in CRC. CAFs carry a broad array of cytokine receptors that allow activation, functional modulation and amplification of effector mechanisms upon signaling events initiated by surrounding tumor cells, immune cells and/or autocrine responses [7,12,13]. CAFs have been shown to shape the TME in CRC and other tumor entities, e.g., by the release of cytokines, growth factors, enzymes and extracellular matrix proteins resulting in multiple consequences influencing the immune system, angiogenesis, tumor mechanics, drug access and therapy responses [12,13,14]. Using an unbiased approach, it was recently suggested that stem cell functionality in CRC is microenvironmentally defined by CAF-derived signals [15]. In addition, paracrine signaling by a mesenchymal niche was reported to orchestrate early development of intestinal tumor providing further evidence for critical role of CAFs in the control of CRC [13]. A major challenge for potential strategies targeting CAFs, however, is the great diversity of the cell type: CAFs are not uniform, but they represent a very heterogenous group with different origin and potent cell plasticity [16]. Various subsets were proposed based on the expression or lack of several molecular markers such as FAP, FSP-1, αSMA, PDGFRβ and type VI collagen [12,16]. Correspondingly, a broad range of different roles was described in tumor biology, e.g., CAFs were demonstrated to fuel tumor development by the release of growth factors, by acting as an immune-suppressive player in the TME or by sustaining cancer stemness promoting cancer formation and chemoresistance [16,17,18,19,20,21]. However, some CAFs were also reported to suppress tumor growth by mechanisms related to immune activation [22]. While they can originate from different sources, it is assumed that CAFs have also a high degree of plasticity [16]. Factors that determine the phenotype and function of CAFs are influenced by the specific cellular and molecular context [9,23,24]. The functional heterogeneity of fibroblast subsets in CRC was exemplified by two studies in which Ikkβ was targeted in COL1+ vs. COL6+ fibroblast populations, resulting in opposing changes in tumor development in the mouse models of colitis-associated CRC [25,26].

Recent work by our group correlated the activation of signal transducer and activator of transcription 3 (STAT3) in CAFs with poor prognosis in CRC [27]. In addition, it was demonstrated that STAT3 activation in type VI collagen-expressing fibroblasts promoted tumor development in experimental in vivo models of CRC [27]. However, the consequence of STAT3 activation in type I collagen-expressing fibroblasts for the tumor growth of CRC in vivo has not been addressed yet. This could be particularly interesting since type I collagen-expressing cells are believed to represent a very frequent CAF population and type I collagen was demonstrated to dominate the extracellular matrix in aggressive CRC [28]. In addition, type I collagen production was connected to a subpopulation of fibroblasts expressing Engrailed-1 (EN1), a transcription factor involved in breast cancer and fibrotic tissue formation [29,30].

Here, we report the role of STAT3 activation in type I collagen-expressing fibroblasts during the growth of colitis-associated CRC, providing evidence for a functional contribution of this CAF subset during tumor development.

## 2. Materials and Methods

### 2.1. Mice

*Col1a2*-CreER^T2^ mice with tamoxifen inducible transgenic expression of the Cre recombinase under the control of the Col1a2 promoter [31] were crossed either with mice bearing a floxed stop cassette followed by a constitutive active STAT3 gene in the ROSA26 locus (*R26Stat3CSTOP^fl/fl^*) [32] or with mice carrying floxed STAT3 alleles (*Stat3^fl/fl^*) [33]. Reporter mice were used expressing tdTOMATO [34] under the control of the inducible Col1a2 promoter *Col1a2*-CreER^T2^ or Col6 promoter *Col6*-Cre, respectively [31,35]. Animal experiments were performed with cohoused littermate controls. The study was in agreement with protocols approved by the governments of Middle Franconia and Rhineland-Palatinate, Germany.

### 2.2. Experimental Tumor Model

The AOM/DSS model was performed to study colitis-associated tumor development with Azoxymethane (Sigma-Aldrich, Saint Louis, MO, USA) and Dextran sulfate sodium salt (MP Biochemicals, Illkirch, France), as described previously [36]. When using tamoxifen inducible strains, Tamoxifen (Cayman Chemical, Ann Arbor, MI, USA) was dissolved in sunflower oil and injected intraperitoneally for five consecutive days one week before each DSS treatment. Each of the three DSS cycles lasted one week and was followed by two weeks of water. Tamoxifen 1 mg was applied in a total volume of 100 µL per injection, and all experimental controls obtained the same treatment.

### 2.3. Mini-Endoscopy

Colorectal tumor development was monitored in vivo using the endoscopic ‘Coloview system’ (Karl Storz), as described before [37,38]. Mice were anesthetized during endoscopy using Isoflurane (Abbott Laboratories, Lake Bluff, IL, USA).

### 2.4. MicroCT

MicroCT imaging was performed using the Quantum FX µCT system (PerkinElmer), as reported before [36]. In brief, mice were anesthetized with Isoflurane and the colon was washed with a disposable pipette filled with H_2_O. An amount of 0.3 mL of the contrast reagent Gastrografin^®^ 100 mg/mL (Bayer, Leverkusen, Germany) was injected intrarectally with a thin flexible tube, and directly afterwards, 0.3 mL of Iomeprol 300 mg/mL (Imeron^®^) was injected. The configuration for imaging was set as follows: 90 kV; 160 μA; FOV: 40 mm; scan time: 2 min. The data were analyzed with ImageJ v1.53c.

### 2.5. Swiss Rolls

Colon tissue including tumors was prepared for Swiss roll analysis as described before [39]. In short, colon tissue was washed with PBS, cut open longitudinally, rolled outside first and attached by a wooden toothpick. Swiss rolls were then fixed in 4% PFA at 4 °C, embedded in paraffin and cut into 4-micrometer-thin sections and stained with hematoxylin and eosin. H&E staining was recorded with a NanoZoomer 2.0-HT slide scanner (Hamamatsu, Higashi-ku, Hamamatsu City, Japan).

### 2.6. Purification of Fibroblasts and CAFs

Colonic fibroblasts were purified as described before [17]. In short, colon tissue was incubated in HBSS (w/o) (Sigma-Aldrich, Saint Louis, MO, USA) containing 2 mM EDTA (Carl Roth, Karlsruhe, Germany), 100 µM EGTA (Carl Roth, Karlsruhe, Germany) and 1% FCS (PanBiotech, Aidenbach, Germany) for 45 min at 37 °C while shaking. The colon pieces were digested with Dulbecco’s modified Eagle medium F-12 (Life Technologies, Carlsbad, CA, USA) containing FCS (4%), 500 µg/mL Collagenase D (Roche, Basel, Switzerland), 500 µg/mL DNase I (Roche, Basel, Switzerland) and 3 mg/mL Dispase II (Roche, Basel, Switzerland). Fibroblasts were cultivated with Dulbecco’s modified Eagle medium F-12 (Life Technologies, Carlsbad, CA, USA) supplemented with 10% FCS (PanBiotech, Aidenbach, Germany), 2.5% HEPES (Sigma-Aldrich, Saint Louis, MO, USA), 1% penicillin/streptomycin (Sigma-Aldrich, Saint Louis, MO, USA), 20 µg/mL Gentamicin sulfate (PanBiotech, Aidenbach, Germany) and 500 µg/mL Amphotericin B (ThermoFisher Scientific, Waltham, MA, USA).

Cancer-associated fibroblasts were purified from colon tumors. In brief, colons were washed with PBS, cut longitudinally and tumors were collected. The tumors were minced manually in small pieces and digested with enzymes of the Lamina Propria Dissociation Kit (Miltenyi, Bergisch Gladbach, Germany) and incubated for 1 h at 37 °C while shaking. With the gentleMACS™ Octo Dissociator (Miltenyi, Bergisch Gladbach, Germany), the tumors were minced 2–3 times. An amount of 500 µL 500 mM EDTA (Carl Roth, Karlsruhe, Germany) was added to the homogenized cell suspension, incubated for 5 min on ice and centrifuged with 1500 rpm for 5 min. Cells were resuspended and plated in Dulbecco’s modified Eagle medium F-12 (Life Technologies, Carlsbad, CA, USA) supplemented with 10% FCS (PanBiotech, Aidenbach, Germany), 2.5% HEPES (Sigma-Aldrich, Saint Louis, MO, USA), 1% penicillin/streptomycin (Sigma-Aldrich, Saint Louis, MO, USA), 20 µg/mL Gentamicin sulfate (PanBiotech, Aidenbach, Germany) and 500 µg/mL Amphotericin B (ThermoFisher Scientific, Waltham, MA, USA).

For long-term cultivation, fibroblasts and CAFs were cultured with Dulbecco’s modified Eagle medium F-12 (Life Technologies, Carlsbad, CA, USA) supplemented with 10% FCS (PanBiotech, Aidenbach, Germany), 2.5% HEPES (Sigma-Aldrich, Saint Louis, MO, USA) and 1% penicillin/streptomycin (Sigma-Aldrich, Saint Louis, MO, USA).

### 2.7. Immunofluorescence

For immunofluorescence frozen (IFF) staining, colon tissue with tdTomato tumors was fixed in 2% PFA + 2% Sucrose for 2 h, embedded in Tissue-Tek^®^ O.C.T.™ Compound (Sakura^®^, AJ Alphen aan den Rijn, The Netherlands), cut into 5 µm thin sections and frozen or directly used for staining. Tissue sections were fixed in 2% PFA + 2% Sucrose for 15 min, washed with PBS and blocked with 10% FCS and 1% bovine serum albumin. The following antibodies were used for IFF: purified rabbit anti-Collagen 1 antibody (polyclonal, abcam, Cambridge, UK) or purified rabbit anti-Collagen 6 antibody (polyclonal, abcam, Cambridge, UK) and the purified rabbit Isotype antibody (polyclonal, BioLegend, San Diego, CA, USA). Antibodies were labelled with the Alexa Fluor™ 647 Antibody Labeling Kit (Thermo Fisher Scientific, Waltham, MA, USA).

For Immunofluorescence paraffin (IFP) staining, a colon tissue was fixed in Rotihistol (Carl Roth, Karlsruhe, Germany), dehydrated and then embedded in paraffin, cut into 4-micrometer-thin sections and incubated at 60 °C for 30 min. Deparaffinization and rehydration were performed by applying Rotihistol for 5 min followed by 100%, 96% and 70% ethanol for 5 min each. This procedure was repeated twice thereafter. For antigen retrieval, Target Retrieval Solution 10× concentrate diluted 1:10 in H_2_O (Agilent Technologies, Santa Clara, CA, USA) was used. The following antibodies were used for IFP staining: eFluor 660-conjugated rat anti-mouse Ki67 (clone: SolA15, eBioscience, San Diego, CA, USA), eFluor 660-conjugated rat IgG2a kappa Isotype antibody (eBioscience, San Diego, CA, USA), AF488-conjugated rat anti-mouse Ep-CAM (clone: G8.8, BioLegend, San Diego, CA, USA), purified rabbit anti-CD3 antibody (clone: CD3-12, Bio-Rad Laboratories GmbH, Feldkirchen, Germany), purified rabbit anti-F4/80 antibody (clone: BM8, eBioscience, San Diego, CA, USA), purified rabbit anti-MPO antibody (polyclonal, abcam, Cambridge, UK) and purified rabbit anti-E-Cadherin antibody (BD Bioscience, Heidelberg, Germany). Secondary antibody donkey anti-rabbit AF647 (BioLegend, San Diego, CA, USA) was used for purified antibodies.

Nuclei were counterstained with Hoechst 33342 (Life Technologies, Carlsbad, CA, USA). Images were acquired with a confocal microscope TCS SP5 II (Leica, Wetzlar, Germany). Quantification was performed with QuPath (Version 0.2.3) [40].

### 2.8. Light Sheet Microscopy

Light sheet fluorescence microscopy (LSFM) was performed as reported before and required sample preparation [41]. In brief, mice were anesthetized using Isoflurane, injected intravenously with 2 µg AF647-conjugated rat anti-mouse CD31 (clone: MEC13.3, BioLegend, San Diego, CA, USA) in PBS. Thirty minutes after injection, mice were euthanized, and the colon was removed and washed with cold PBS. Colon tissue with tumors was cut into 1 cm pieces and fixed with 2% PFA/PBS for 30 min. After fixation, the colon pieces were washed with cold PBS and afterwards dehydrated with 50%, 70% and 100% ethanol over 3 days. On day 3, colon pieces were transferred to ethyl cinnamate (ECi) (Sigma-Aldrich, Saint Louis, MO, USA) and incubated for 2 to 3 h while gently shaking at room temperature until the tissue was cleared completely. Then, pictures of the samples were acquired in Z-stacks with a LaVision UltraMicroscope II Light Sheet Microscope (LaVision BioTec GmbH, Bielefeld, Germany). Analyses and 3D reconstruction of LSFM data were performed with Fiji/ImageJ (Version 1.53c) and customized ImageJ macros. A 3D animation of LSFM data was produced, as described before [42].

### 2.9. Statistical Analysis

Quantitative data are displayed as mean values per group with error bars showing standard deviations. For statistical analysis, the nonparametric two-tailed Mann–Whitney U test for comparisons of two groups were performed with GraphPad Prism V.8.3.0. Significant differences are indicated as * *p* ≤ 0.05 and ** *p* ≤ 0.01.

## 3. Results

### 3.1. Light Sheet Fluorescence Microscopy of Reporter Mice Demonstrates a Dense Network of Type I Collagen-Expressing CAFs in Experimental CRC

**Figure 1:**
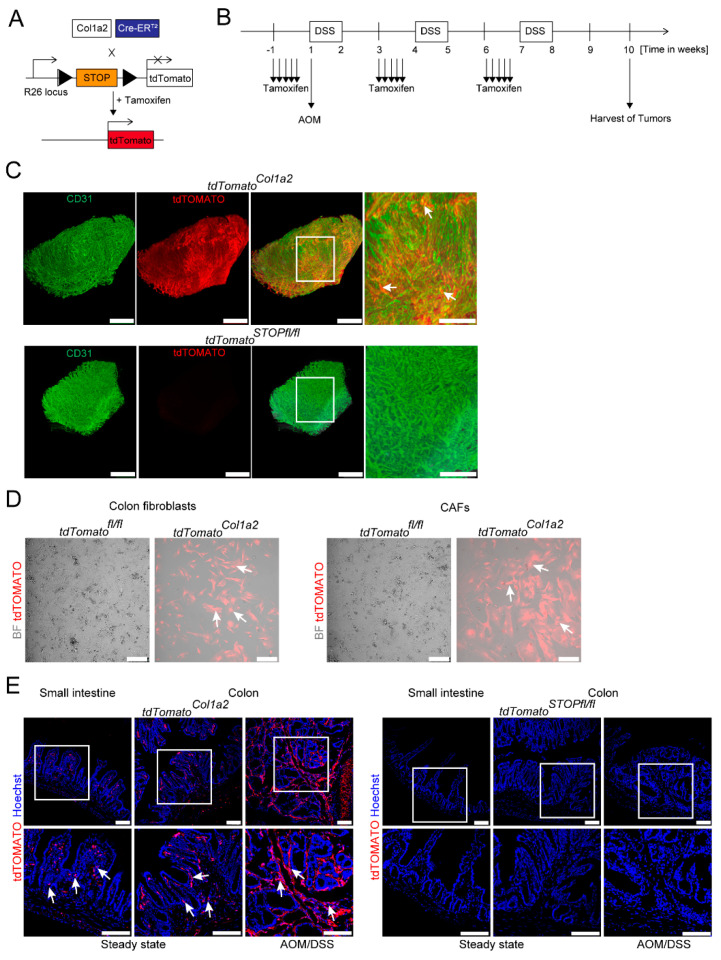
Intestinal fibroblasts and CAFs of the AOM/DSS model are labeled by *tdTomato^Col1a2^* reporter mice and can be visualized by light sheet and confocal fluorescence microscopy. (**A**) Schematic illustration of the genetic approach based on the Cre/loxP system to trace COL1+ fibroblast by cross-breeding *Col1a2*-CreER^T2^ and *tdTomato^STOPfl/fl^* mice. Triangles represent loxP sites. (**B**) Timeline for the experimental setup of the AOM/DSS model using the tamoxifen inducible Col1a2 promoter. (**C**) Colon tumor tissue was harvested from the AOM/DSS model from *tdTomato^Col1a2^* and *tdTomato^STOPfl/fl^* control mice, prepared by ECi-based tissue clearing and analyzed by light sheet fluorescence microscopy. Individual and combined stainings of tdTOMATO (red) and CD31 (green) are shown as indicated in the 3D reconstruction. Scale bar: left 500 µm and right 250 µm. Arrows point to tdTOMATO+ cells. (**D**) Colon fibroblasts and CAFs from the AOM/DSS model were purified from *tdTomato^Col1a2^* and *tdTomato^STOPfl/fl^* control mice, as indicated and studied by fluorescence microscopy. Scale bar: 100 µm. (**E**) Tissue sections were prepared from the small intestine, the colon or AOM/DSS-induced colon tumors from *tdTomato^Col1a2^* and *tdTomato^STOPfl/fl^* control mice and analyzed by confocal fluorescence microscopy as labeled. Arrows indicate tdTOMATO+ cells. Scale bar: 100 µm. Pictures in (**C**–**E**) are representative of at least two independent experiments.

Previous studies by others suggested opposing roles of type I vs. type VI collagen-expressing fibroblasts in the AOM/DSS model of CRC [25,26]. Recent work by our group has demonstrated a critical contribution of STAT3 activation in type VI collagen-expressing fibroblasts in CRC [27]. Here, we focused on the role of STAT3 activation in the subset of type I collagen-expressing fibroblasts. Therefore, we took advantage of a genetically modified mouse strain expressing the Cre recombinase under control of the Col1a2 promoter (*Col1a2*-CreER^T2^). To validate this approach and to demonstrate the spatial distribution of type I collagen-expressing fibroblasts in experimental CRC in our setup, reporter mice (*tdTOMATO^Col1a2^*) expressing red fluorescence protein tdTOMATO [34] were generated using the Cre/loxP system (Figure 1A). Next, these mice were applied to the AOM/DSS model (Figure 1B), and colon tissue was analyzed by light sheet microscopy upon staining for CD31. Hereby, we could visualize that such tumors have a rich vasculature and that their stroma is populated massively by tdTOMATO+ cells indicating a high number of type I collagen-expressing CAFs forming a dense network in TME (Figure 1C).

Efficient targeting of CAFs was confirmed by brightfield microscopy of single cell fibroblasts purified from AOM/DSS colon tumors. Here, the relative frequency of tdTOMATO targeted fibroblasts and CAFs was routinely around 80% (Figure 1D), confirming the assumption that collagen I is a reliable marker for the majority of CAFs in this model, which is in line with previous reports [25]. In addition, fibroblasts purified from unchallenged colon tissue were also targeted as evaluated by brightfield microscopy (Figure 1D).

Further ex vivo analysis by immunofluorescence confirmed the expression of tdTOMATO in fibroblasts of small and large intestines during steady-state conditions and the accumulation of tdTOMATO+ CAFs in AOM/DSS-induced tumors (Figure 1E).

### 3.2. Constitutive Activation of STAT3 in Type I Collagen-Expressing Fibroblasts Enhances Colorectal Carcinogenesis

**Figure 2:**
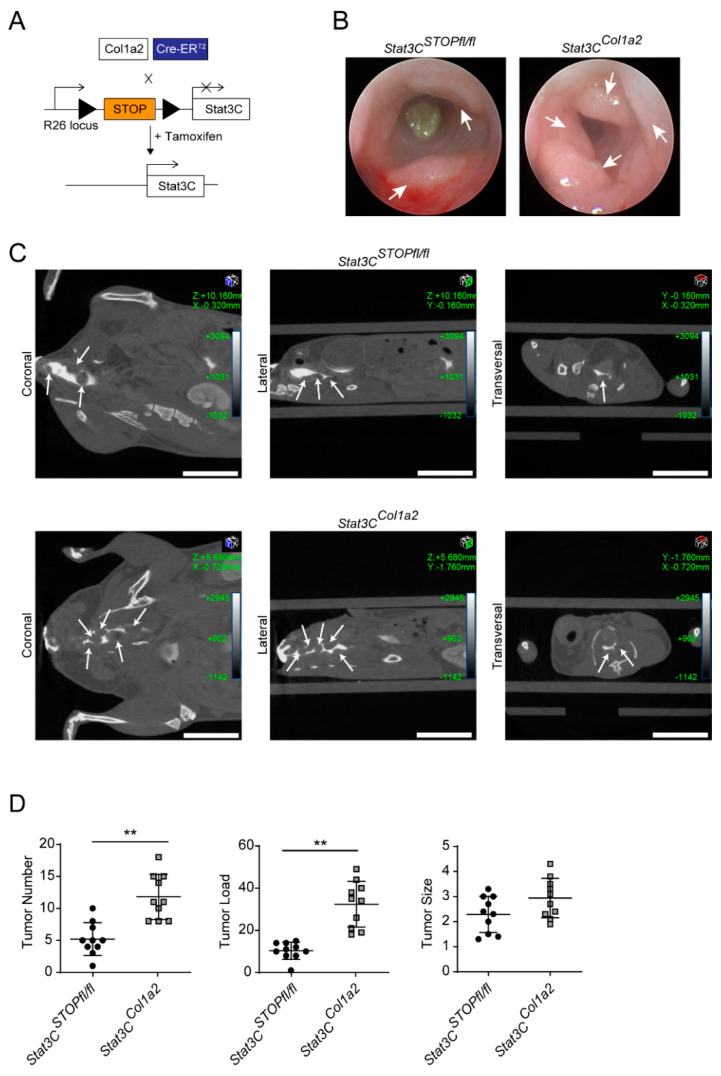
Constitutive activation of STAT3 in type I collagen-expressing fibroblasts promotes the development of CRC in the AOM/DSS model. (**A**) Scheme of the genetic strategy for the expression of a constitutively active STAT3 (STAT3C) protein in COL1+ cells by cross-breeding of *Col1a2*-CreER^T2^ with *R26Stat3C^STOPfl/fl^* mice. Triangles show loxP sites. (**B**) Tumor development in the AOM/DSS model was monitored in *Stat3C^Col1a2^* mice and littermate controls by mini-endoscopy at day 62. (**C**) Tumor development in the AOM/DSS model was analyzed in *Stat3C^Col1a2^* mice and littermate controls by contrast-agent-enhanced micro-CT and shown in coronal, sagittal and axial planes. Arrows indicate colon tumors. Scale bar: 10 mm. (**D**) Tumor number, tumor load and tumor size (mm) of AOM/DSS-treated *Stat3C^STOPfl/fl^* (*n* = 10) and *Stat3C^Col1a2^* mice (*n* = 10) were quantified ex vivo at day 64. The results are representative of at least two independent experiments. Quantitative data are shown as mean values ± SD. Significant differences are indicated (** *p* < 0.01).

**Figure 3:**
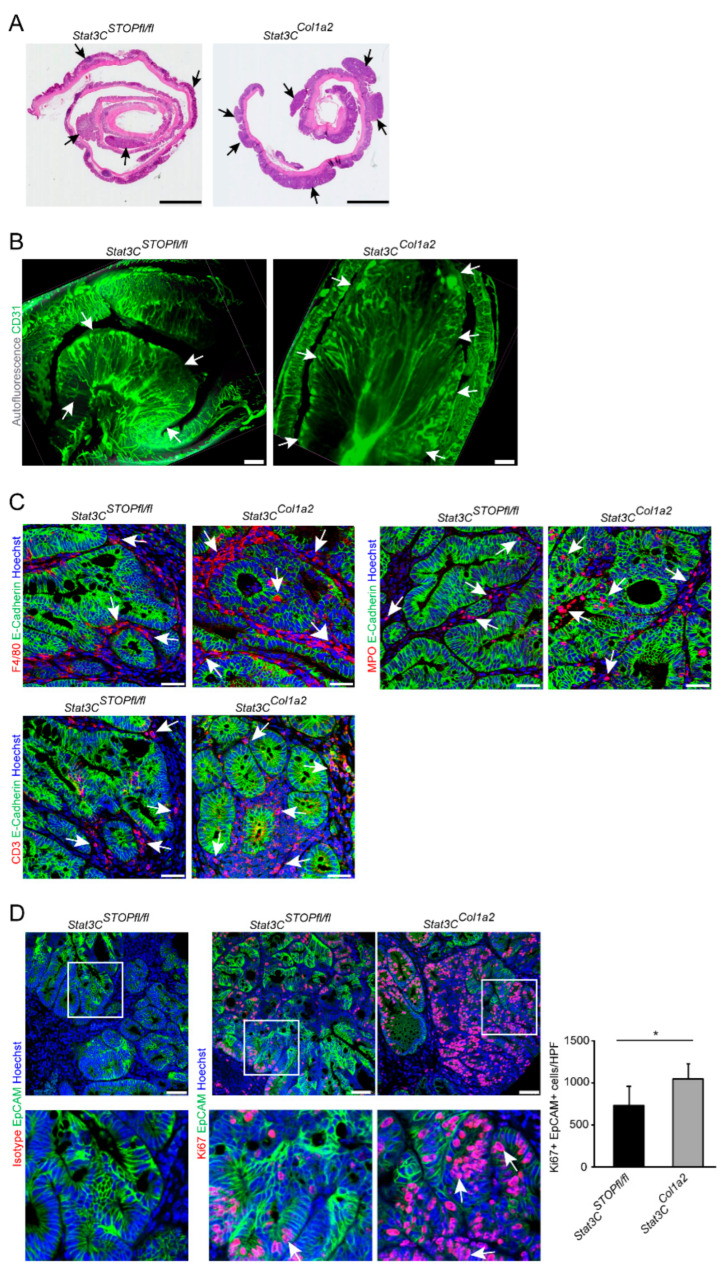
Constitutive activation of STAT3 in type I collagen-expressing fibroblasts increases tumor cell proliferation. (**A**) Colon tissue with tumors from AOM/DSS-treated *Stat3C^Col1a2^* mice and littermate controls was prepared as Swiss rolls, stained by H&E and analyzed by microscopy. Arrows indicate tumors. Scale bar: 2500 µm. (**B**) Colon tumors of the AOM/DSS model from *Stat3C^Col1a2^* mice and littermate controls were stained with a fluorescently labeled anti-CD31 antibody (green) and visualized by light sheet fluorescence microscopy. Autofluorescence is shown in grey. Arrows highlight tumors. Scale bar: 500 µm. (**C**) AOM/DSS tumors were harvested from *Stat3C^Col1a2^* mice and *Stat3C^STOPfl/fl^* controls at day 64 and analyzed by immunofluorescence staining for immune cells (F4/80+, MPO+ or CD3+) and E-Cadherin as indicated. Arrows indicate infiltrating cells. Scale bar: 50 µm. (**D**) AOM/DSS tumor tissue (day 64 from *Stat3C^Col1a2^* mice and *Stat3C^STOPfl/fl^* controls (*n* = 4–7/group) was immunofluorescently co-stained for Ki67 and EpCAM and analyzed by confocal microscopy. For each sample, 45 representative HPFs were scored. Arrows highlight Ki67+EpCAM+ tumor cells. Scale bar: 50 µm. The results are representative of at least two independent experiments. Quantitative data are shown as mean values ±SD. Significant differences are indicated (* *p* < 0.05)).

To evaluate the contribution of STAT3 activation in type I collagen-expressing fibroblasts during the development of experimental CRC, we crossbred *Col1a2*-CreER^T2^ with *R26Stat3C^STOPfl/fl^* mice [32]. Hereby, we obtained animals expressing a constitutively active STAT3 (STAT3C) protein in type I collagen-expressing fibroblasts (*Stat3C^Col1a2^* mice, Figure 2A). *Stat3C^Col1a2^* mice did not show any phenotypical abnormalities under steady-state conditions (data not shown). Next, we studied the formation of colorectal tumors in mice challenged with AOM/DSS. Interestingly, we observed increased tumor development in *Stat3C^Col1a2^* mice as compared to littermate controls evaluated by mini-colonoscopy (Figure 2B). This finding was fortified when mice were dissected by CT scans (Figure 2C). In addition to in vivo imaging and analysis, ex vivo quantification assaying the tumor number and tumor load at the freshly harvested colon macroscopically confirmed previous findings. The mice were sacrificed immediately after the last DSS cycle because of the increased tumor burden in *Stat3C^Col1a2^* mice. In fact, mice with a constitutively active STAT3 protein revealed a highly significant change with clearly enhanced tumorigenesis as compared to controls (Figure 2D), although there was no evidence for invasive tumor growth or metastasis. In addition, histopathological analysis of H&E-stained Swiss roll preparations demonstrated the presence of numerous large colorectal tumors in *Stat3C^Col1a2^* mice (Figure 3A), which was also confirmed by LSFM, further suggesting a strong vascularization of the tumors as evidenced by staining of CD31 (Figure 3B). Next, we analyzed the presence of inflammatory cells in AOM/DSS tumor tissue from the different experimental conditions. Here, we detected a strong infiltration of immune cells as evaluated by immunofluorescence stainings with markers for innate and adaptive immune cells (MPO, F4/80, CD3). Although we did not detect major differences, there was a trend towards a higher number of immune cells in tumors from *Stat3C^Col1a2^* mice as compared to controls (*Stat3C^STOPfl/fl^*) for markers F4/80 and CD3 but not for MPO (Figure 3C). Moreover, we observed an increased number of Ki67+ EpCAM+ cells highlighting enhanced proliferation of tumor epithelial cells in cancer tissue of *Stat3C^Col1a2^* mice as compared with littermate controls (Figure 3D).

Hypoxia-related mechanisms could potentially modulate tumor development in our setup as supported by a trend towards the increased expression of Sox2, Tert, Pou5f1 and Cxcr4 in AOM/DSS tumors from *Stat3C^Col1a2^* mice, but we did not detect a direct regulation of HIF by STAT3 in purified COL1+ fibroblasts in an oxygen-independent manner. Elevated expression of Engrailed-1 was observed in tumors from *Stat3C^Col1a2^* mice as compared to tumors from controls.

Thus, a gain-of-function approach using mice with a constitutively active STAT3 protein in type I collagen-expressing fibroblasts suggests a critical role of STAT3-related signaling in this subset of cells to drive tumor growth by inducing the enhanced proliferation of tumor epithelial cells in inflammation-associated CRC models.

### 3.3. STAT3 Inactivation in Type I Collagen-Expressing Fibroblasts Reduces Experimental CRC

**Figure 4:**
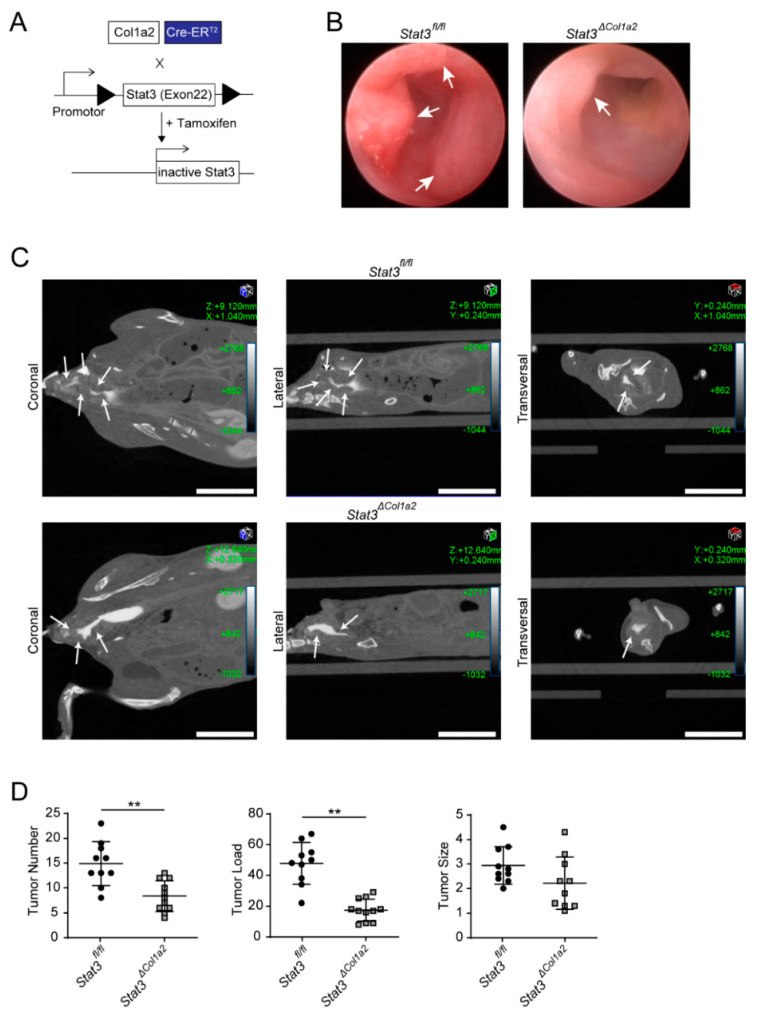
STAT3 inactivation in type I collagen-expressing fibroblasts reduces inflammation-associated experimental tumorigenesis. (**A**) Schematic illustration of the Cre/loxP-based approach to conditionally target STAT3 inactivation in COL1+ cells by cross-breeding of *Col1a2*-CreER^T2^ with *Stat3^fl/fl^* mice. Tamoxifen-induced expression of Cre recombinase results in the deletion of exon 22 (encoding the tyrosine residue (Y705) critical for STAT3 activation), resulting in a STAT3 protein that can no longer be activated. Triangles highlight loxP sites. (**B**) Tumorigenesis in the AOM/DSS model was analyzed in *Stat3*^Δ*Col1a2*^ mice and *Stat3^fl/fl^* controls by mini-endoscopy at day 62. (**C**) Tumor growth in the AOM/DSS model was studied in *Stat3*^Δ*Col1a2*^ mice and littermate controls by contrast-agent-enhanced micro-CT and presented in coronal, sagittal and axial planes. Arrows point to colon tumors. Scale bar: 10 mm. (**D**) Tumor number, tumor load and tumor size (mm) of AOM/DSS-treated *Stat3*^Δ*Col1a2*^ mice (*n* = 11) and littermate controls (*n* = 12) were measured ex vivo at day 64. The results are at least of two independent experiments. Quantitative data are shown as mean values ± SD. Significant differences are indicated (** *p* < 0.01).

Next, we pursued a loss-of-function strategy and tested the consequence of STAT3 inactivation in type I collagen-expressing fibroblasts. Therefore, we crossed *Col1a2*-CreER^T2^ mice with genetically modified mice carrying floxed STAT3 alleles [33] (Figure 4A).

**Figure 5:**
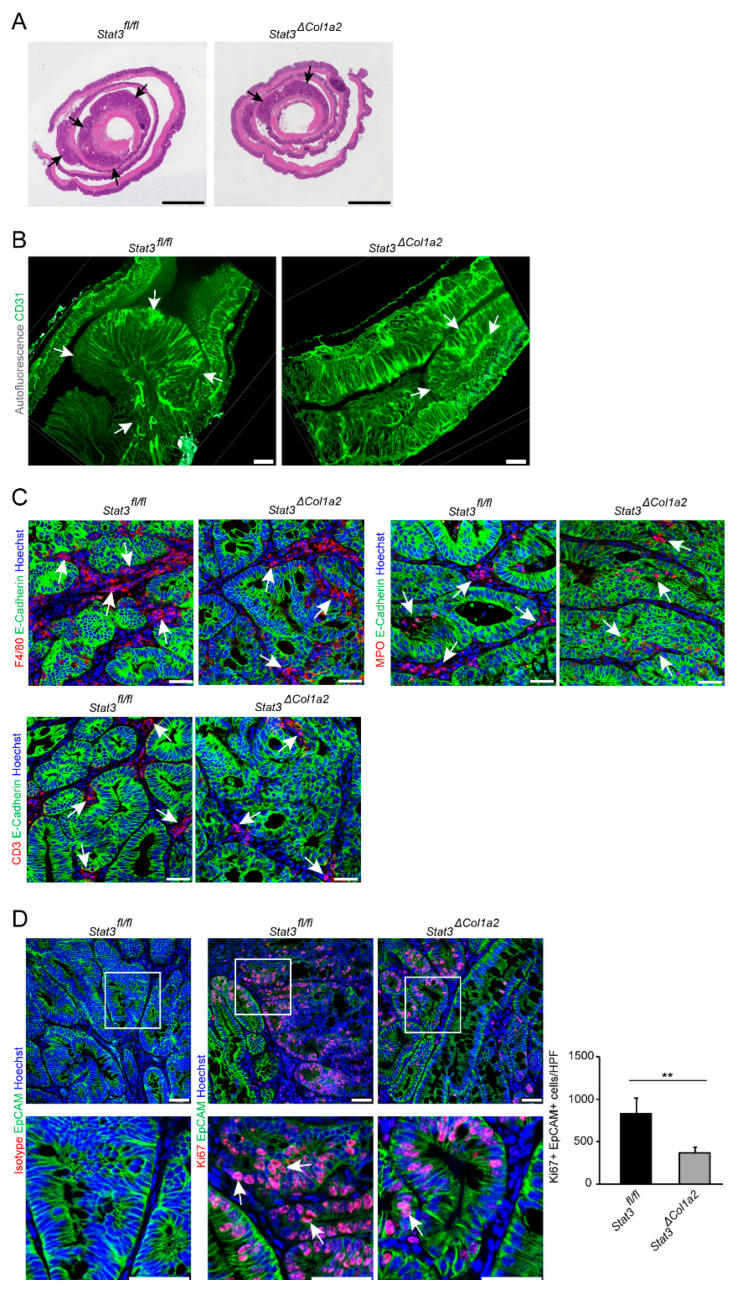
STAT3 inactivation in type I collagen-expressing fibroblasts decreases tumor cell proliferation. (**A**) Colon tissue with AOM/DSS-induced tumors from *Stat3*^Δ*Col1a2*^ mice and littermate controls was prepared as Swiss rolls, stained by H&E and examined by microscopy. Arrows indicate tumors. Scale bar: 2500 µm. (**B**) Colon tumors of the AOM/DSS model from *Stat3*^Δ*Col1a2*^ mice and littermate controls were stained with a fluorescently labeled anti-CD31 antibody (green) and visualized by light sheet fluorescence microscopy. Autofluorescence is shown in grey. Arrows highlight tumors. Scale bar: 500 µm. (**C**) AOM/DSS tumors were obtained from *Stat3*^Δ^*^Col1a2^* mice and *Stat3^fl/fl^* controls at day 64 and stained for immune cell markers (F4/80+, MPO+ or CD3+) and E-Cadherin as indicated. Arrows indicate infiltrating cells. Scale bar: 50 µm. (**D**) AOM/DSS tumor tissue (day 64) from *Stat3*^Δ*Col1a2*^ mice and *Stat3^fl/fl^* controls (*n* = 3–4/group) was labeled by antibodies for Ki67 and EpCAM and analyzed by confocal fluorescent microscopy. For each sample, 4–5 representative HPFs were quantified. Arrows point to Ki67+EpCAM+ tumor cells. Scale bar: 50 µm. The data are representative of at least two independent experiments. Quantitative data are shown as mean values ± SD. Significant differences are indicated (** *p* < 0.01).

Then, the AOM/DSS model was applied to these mice (*Stat3*^Δ*Col1a2*^) and inflammation-driven tumor development was monitored in vivo and compared with littermate controls. Strikingly, tumor growth was clearly reduced in *Stat3*^Δ*Col1a2*^ as analyzed by mini-colonoscopy (Figure 4B) and CT scan (Figure 4C). Similar findings were observed when the tumor number and the tumor load were quantified ex vivo by macroscopic inspection at freshly harvested colons (Figure 4D). Histopathological analysis of Swiss rolls stained with H&E showed a reduced number of tumors from *Stat3*^Δ*Col1a2*^ mice as compared to tumors from littermate controls (Figure 5A). In addition, colorectal tumors from *Stat3*^Δ*Col1a2*^ and corresponding littermate controls were analyzed ex vivo by advanced imaging with LSFM, which included the visualization of CD31+ blood vessels in TME (Figure 5B). Subsequently, we studied the infiltration of immune cells in AOM/DSS tumor tissue in our setup. Again, we detected a strong infiltration of immune cells, as evaluated by immunofluorescence staining with markers for innate and adaptive immune cells (MPO, F4/80, CD3). However, we observed a clear trend towards a diminished number of immune cells in tumors from *Stat3*^Δ*Col1a2*^ mice as compared to the controls (*Stat3^fl/fl^*) for markers F4/80 and CD3 but not for MPO (Figure 5C). Finally, we addressed the proliferation of tumor epithelial cells by IF-staining for Ki67 and EpCAM. Here, we detected a reduced number of Ki67+ EpCAM+ cells in *Stat3*^Δ*Col1a2*^ mice as compared with littermate controls (Figure 5D), indicating the reduced proliferation of tumor epithelial cells in mice with conditional STAT3 inactivation in type I collagen-expressing fibroblasts.

Thus, these results provide further evidence that STAT3 activation in type I collagen-expressing fibroblasts modulates tumor epithelial cells and drives CRC development in the AOM/DSS model.

### 3.4. Type I Collagen-Expressing Fibroblasts Include a Subpopulation of Type VI Collagen-Expressing Fibroblasts in the AOM/DSS Model and the Unchallenged Colon

**Figure 6:**
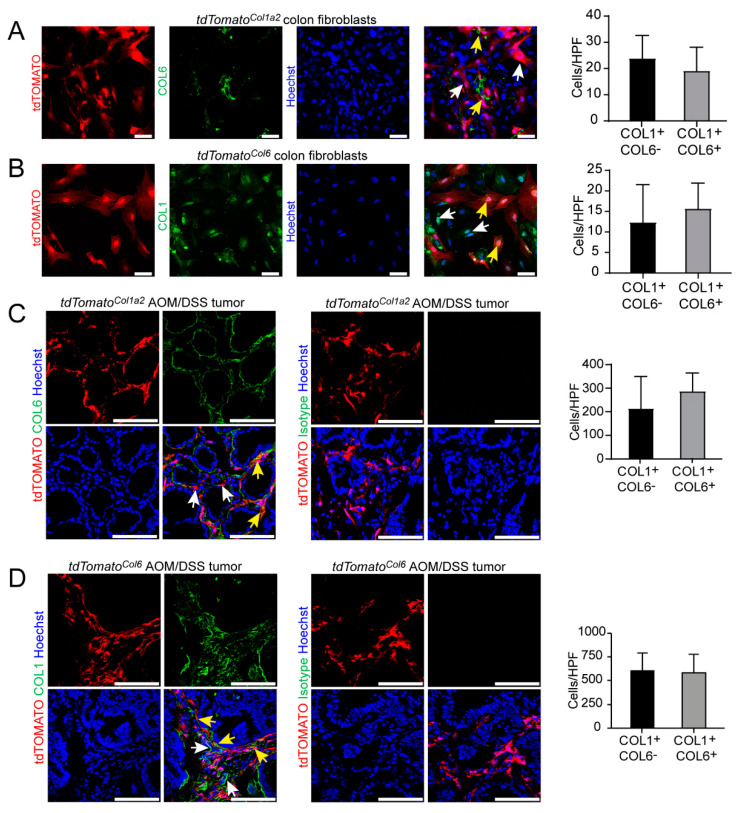
COL1+ fibroblasts represent a main fibroblast population and include a COL6+ subset in the unchallenged colon and the AOM/DSS model. (**A**) Colon fibroblasts were isolated from Tamoxifen administered *tdTomato^Col1a2^* reporter mice with enzyme-based purification, stained with anti-Collagen 6 (green) and Hoechst (blue) and studied by confocal microscopy. For each sample, 4–5 representative HPFs were scored for the number of COL1+COL6+ (yellow arrows) and COL1+COL6- (white arrows) cells (*n* = 4/group). Scale bar: 50 µm. (**B**) Colon fibroblasts were purified from *tdTomato^Col6^* reporter mice, plated on chamber slides, stained with anti-Collagen 1 antibody (green) and Hoechst (blue) and examined by confocal microscopy. For each sample, 4–5 representative HPFs were scored for the number of COL1+COL6+ (yellow arrows) and COL1+COL6- (white arrows) cells (*n* = 4/group). Scale bar: 50 µm. (**C**) Tissue sections of AOM/DSS tumors from *tdTomato^Col1a2^* reporter mice were stained with immunofluorescently labeled anti-Collagen 6 or isotype control antibodies (green) and Hoechst (blue). For each sample, 4–5 representative HPFs were quantified for the number of COL1+COL6+ (yellow arrows) and COL1+COL6- (white arrows) cells (*n* = 3/group). Scale bar: 100 µm. (**D**) Tumor tissue of AOM/DSS tumors from *tdTomato^Col6^* reporter mice were stained with anti-Collagen 1 or isotype control antibodies (green) and Hoechst (blue) and analyzed by confocal fluorescent microscopy. For each sample, 4–5 representative HPFs were studied for the number of COL1+COL6+ (yellow arrows) and COL1+COL6- (white arrows) cells (*n* = 3/group). Scale bar: 100 µm. The results are representative of at least two independent experiments.

Our findings on the role of STAT3 activation in type I collagen-expressing fibroblasts were similar to recent observations on the contribution of STAT3 activation in type VI collagen-expressing fibroblasts [27]. In view of previous data that had demonstrated opposing functions of these fibroblast subsets upon Ikkβ inactivation, we wondered about the potential overlap between COL1+ and COL6+ cells in our setup. To address this question, we planned to analyze the co-expression of type I and type VI collagen-producing cells. However, reliable antibody-based staining of different collagens in the same sample preparations was challenging. To avoid potential interactions between antibodies, we took advantage of reporter mice expressing the red fluorescent protein tdTOMATO under the control of the Col1 promoter (*tdTomato^Col1a2^*) or the Col6 promoter (*tdTomato^Col6^*), respectively. Fibroblasts were purified from the colon of unchallenged *tdTomato^Col1a2^* reporter mice, stained with a COL6-directed antibody and analyzed by immunofluorescence confocal microscopy. We observed that the great majority of the cells were positive for tdTOMATO (i.e., COL1), whereas about half of the cells were positive for COL6 (Figure 6A) indicating two major populations, COL1+COL6- and COL1+COL6+, with a similar frequency. These data suggested that the COL6+ population represented mainly a subset of type I collagen-expressing fibroblasts (Figure 6A). Next, a reverse setup was used in which fibroblasts were purified from the colon of *tdTomato^Col6^* reporter mice and stained with a COL1-directed antibody (Figure 6B). Analysis by immunofluorescence confocal microscopy revealed similar results demonstrating two major subpopulations (namely COL1+COL6- and COL1+COL6+), whereas there was no clear evidence for a substantial proportion of COL1-COL6+ cells (Figure 6B). Thereafter, we studied tumor tissues from the AOM/DSS model addressing the co-expression of type I and type VI collagen-producing CAFs. Cross sections of colorectal tumors of *tdTomato^CoI1a2^* mice were immunofluorescently labeled with anti-Collagen 6 or isotype control antibody, respectively. Although both type I collagen (i.e., tdTOMATO)-expressing cells as well as type VI collagen-expressing cells were abundantly expressed in the TME of AOM/DSS tumors, and the COL1+ population represented the majority of CAFs (Figure 6C). Again, we observed two major CAF subsets (COL1+COL6- and COL1+COL6+) of similar frequencies, but we failed to obtain evidence for a prominent COL1-COL6+ subpopulation in our analysis (Figure 6C). Next, the reverse setup was also applied using *tdTomato^Col6^* reporter mice and immunofluorescence staining with a COL1-directed antibody, which confirmed our previous findings (Figure 6D). Thus, our study demonstrates that type I collagen-expressing cells represent the majority of fibroblasts and include a subpopulation of type VI collagen producing fibroblasts in both the AOM/DSS model and the unchallenged colon. In addition, our own investigations into the activation status of different pathways confirmed a high frequency of active STAT3 and active NFκB in both COL1+ CAFs and COL6+ CAFs in AOM/DSS tumors.

In summary, our data highlight a critical role of STAT3 activation in type I collagen-expressing fibroblasts for the growth of experimental colorectal cancer in the AOM/DSS model. COL1+ cells constitute the majority of CAFs in TME, and they can be divided in two similarly sized subsets with or without the co-expression of type VI collagen.

## 4. Discussion

The importance of the tumor microenvironment (TME) as a major player of the tumor biology in CRC has been increasingly recognized and the prognostic value was demonstrated for markers associated with the tumor stroma in CRC [6,9,27,43]. Our work indicates that pSTAT3 activation in type I collagen-expressing fibroblasts controls tumor development in colitis-associated cancer as evidenced by in vivo data with genetically modified mice in a gain-of-function and a loss-of-function approach, respectively. Hence, our study adds to the growing body of evidence that CAFs and other stromal cells influence adjoining cancer cells and modulate tumor development [2].

Our current work focused on the role of STAT3 activation (in COL1+) fibroblasts in the AOM/DSS mouse model mimicking colitis-associated CRC, which is a well-known long-term complication of chronic colitis in inflammatory bowel diseases [2]. Colitis-associated CRCs typically show some characteristic morphological features and a rapid growth speed, which are believed to be driven by various molecular mechanisms including a different sequence of mutations in key tumor suppressor genes, the high abundance of pro-inflammatory cytokines in the TME and the release of CAF-derived growth factors [2,17,26,44,45]. Nevertheless, we and others provided evidence previously that CAFs can substantially modulate the development of sporadic CRC as well [9,27]. However, future studies are needed to further delineate potential roles of COL1+ fibroblasts in sporadic CRC specifically.

The characterization and functional analysis of CAF subsets is a current focus of research, e.g., single-cell transcriptomic analysis of human CRC demonstrated cellular heterogeneity and reported that CAFs might comprise two major subtypes (CAF-A and CAF-B) with distinct transcriptomic profiles [44]. Notably, COL1A2 was one of the marker genes in addition to MMP2 and DCN for the CAF-A subtype, which might suggest that our findings are related primarily to STAT3 modulation of rather CAF-A than CAF-B type of cells [44]. Thus, the findings presented here correspond with other studies suggesting phenotypical and functional heterogeneity among fibroblasts in CRC. In addition, it was recently reported that COX-2/Ptgs2/PGE2 expressing Pdgfr^low^ pericryptal fibroblasts guide intestinal tumorigenesis in mice in a paracrine manner by promoting early lesions of CRC [13]. Conversely, our data and recent studies by others suggest that the activation of specific CAF subpopulations can also support the development of tumor progression at later stages [17,25,26,27]. In addition, CAFs are considered to exhibit plasticity that is influenced by paracrine and autocrine signaling within the tumor stroma in CRC and other tumor types [24,27].

The findings presented here demonstrate that pSTAT3 activation in type I collagen-expressing fibroblasts drives tumor development in the AOM/DSS model. In addition, our findings linked STAT3 activation in COL1+ CAFs with enhanced proliferation in tumor epithelial cells, suggesting paracrine and/or contact dependent molecular cross-talk.

It seems possible that hypoxia-related mechanisms also influenced the tumor development in our study, although we did not observe a direct regulation of HIF by STAT3 in COL1+ fibroblasts in an oxygen-independent manner. Correspondingly, STAT3 was reported to modulate hypoxia-induced mechanisms in other tumor entities [46,47]. Additional mouse strains, e.g., with a constitutive STAT3 activation in Col1+ fibroblasts on a HIF1 deficient background, could be helpful for determining the contribution of HIF-dependent tumor progression in more detail in our setup. Our current data correspond with previous work by others reporting that type I collagen dominates the extracellular matrix in aggressive CRC [28]. As the AOM/DSS model allows studying the sequential development of colon carcinogenesis, it could be interesting to compare the consequence of the inducible COL1-specific activation/inactivation of STAT3 at different time points during the AOM/DSS protocol in future studies.

Moreover, we observed that COL1+ CAFs can be divided into two similarly sized subsets (COL1+ COL6+ and COL1+ COL6-), whereas we did not detect any major fraction of COL6+COL1- CAFs, suggesting that COL6+ CAFs represent a subpopulation of COL1+ CAFs. Notably, genetically modified mouse strains such as Col1Cre or Col6Cre are frequently used to tackle questions about the role of fibroblasts in experimental models and to conditionally target CAF subsets in CRC [13,25,26]. Interestingly, opposing functions were reported by using the AOM/DSS model in previous studies for the selective inhibition of IKKβ in COL1+ or COL6+ fibroblasts, respectively [25,26]. By contrast, our data indicate a similar role for STAT3 activation in both CAF subtypes. In fact, tumor development was reduced in each case when STAT3 was inhibited in the population of COL1+ or COL6+ fibroblasts, respectively (this study and [27]). Corresponding results were found in experiments exploring the functional significance of type I or type VI collagen-expressing fibroblasts using the gain of STAT3 function approaches (this study and [27]). Hence, the current work adds interesting information on the already multifaceted picture of CAFs in CRC, and the aforementioned studies altogether highlight the complexity of fibroblast heterogeneity and its functional implications during colitis-associated carcinogenesis. Accordingly, it appears remarkable that defined CAF subsets might act synergistically or antagonistically depending on the signaling pathway activated, e.g., upon activation of STAT3 vs. NFκB. Moreover, it seems particularly interesting as the data presented here in a model of colitis-associated tumorigenesis indicate that COL6+ CAFs represent rather a subset of COL1+ CAFs than a fully distinct population. Thus, it could be interesting to address the molecular conditions that further define the agonistic vs. antagonistic functions of specific CAF subsets in the stroma of CRC and other tumor diseases.

Nevertheless, it is well known that tumor development in CRC is only partly influenced by genetic cues. In addition, environmental factors, e.g., the composition of the microbiome and chow, or disparities in the experimental protocols are also critical variables. Such influences can represent potential confounders when studies performed at different locations are compared to each other [36,45]. Thus, additional studies are required to better clarify the functional network guided by different CAF subsets in the TME of CRC. Notably, novel mouse models based on NOTCH activation in tumor cells were recently reported, recapitulating the important features of mesenchymal CRC (CMS4) [48,49]. Such additional model systems might serve as valuable tools to further decipher the molecular cross-talk and cell plasticity within the TME, including the specific contributions from CAFs, e.g., by secretion of soluble factors, matrix remodeling and metabolic effects possibly associated with STAT3 activation.

Evidence from different tumor entities suggested that the inhibition of STAT3-associated signaling could represent a suitable therapeutic approach. In studies addressing the significance of STAT3 in CRC, STAT3 activation in different cell types including tumor cells and myeloid cells has been connected to the promotion of tumor development [50,51,52]. In addition, epithelial STAT3 was reported to correlate with unfavorable outcomes in human CRC [53]. Moreover, the potential clinical relevance is underlined by recent observations from our group, showing that increased STAT3 activation in fibroblasts in human CRC at early tumor stages is associated with reduced overall survival [27]. Therapeutic testing using STAT3 inhibitors have demonstrated promising results in some tumor diseases [54,55]. The results of this study argue for the potential importance of STAT3 signaling as a therapeutic target in CRC, which corresponds with previous work by others and us [27,50,56]. However, this concept was recently challenged by the results of a randomized phase 3 trial showing that the application of the STAT3 inhibitor napabucasin in a patient cohort with therapy-refractory metastatic CRC did not meet the primary endpoint (overall survival) in an intention-to-treat analysis [57]. Nevertheless, the interpretation of the study needs some caution and subgroup analyses suggested that STAT3 could still serve as potential target, especially for CRC with high pSTAT3 expressions in IECs [57]. In addition, it is tempting to speculate whether additional criteria, such as tumor subtype, tumor stage or molecular features of the stroma, e.g., pSTAT3^high^ in CAFs or CMS type IV (which both correlate with poor prognosis), might aid in enhancing precision and success in therapeutic approaches targeting STAT3 activation in CRC [9,11,27]. On the basis of our results, strategies blocking STAT3 activation in defined CAF populations of colitis-associated CRC could be promising. However, powerful tools that could allow for specific molecular targeting of CAFs in human CRC in clinical trials are still lacking.

## 5. Conclusions

In summary, our investigations revealed a critical role of STAT3 activation in COL1+ CAFs, suggesting that targeting of STAT3 in type I collagen-expressing fibroblasts could represent a promising therapeutic strategy in colitis-associated CRC and possibly other tumor entities. Our work—in combination with previous studies by others and us—supports a picture in which context-dependent contributions by COL1+ CAFs and COL6+ CAFs might be variable between both populations depending on the signaling pathway activated.

## Data Availability

The data presented in this study are available in this article.
